# Health and Welfare of Captive Reptiles, Second Edition

**DOI:** 10.1017/awf.2023.92

**Published:** 2023-11-06

**Authors:** Frances M. Baines

**Affiliations:** UV Guide UK Abergavenny, Wales, UK

I was delighted to be asked to review this book, having recently retired from many years working as educator and advisor on reptile husbandry with a strong emphasis on improving welfare, in both professional zoological and ‘hobbyist’ communities, and also as a long-term keeper of reptiles myself. *Health and Welfare of Captive Reptiles, Second Edition* is a huge 19-chapter, 638-page book which sells at just under £200; I doubt it will feature on many private reptile-keepers’ bookshelves. It is not, as its title might suggest, a simple manual on how to improve reptile health or welfare; instead, it consists of a series of very detailed literature reviews on aspects of reptile physiology and behaviour, and detailed discussions on the challenges these create for those hoping to give these sentient beings a “life worth living”. Each chapter is heavy with references. Frustratingly, a high proportion of these are merely cross-references to other chapters in the book, and many others are inaccessible without access to an institutional library. However, inclusion of the weblinks to those which are freely available in the reference lists is a welcome touch.

I suspect the book will prove most valuable to educators in veterinary schools and agricultural colleges which include exotic animal husbandry courses, who do need to include an appreciation of reptile sentience and welfare assessments in their teaching material, and to students in these colleges, broadening their specialist knowledge on the problems facing captive reptiles. Many of these are unique to ectotherms owing to their need for enclosures with controlled environments when housed within human habitation.

Those involved in education of the pet-keeping public on social media, or via other means such as websites, workshops, lectures, or writing popular books and magazines would also do well to study many of the chapters within. Much of the information also needs to be shared with zookeepers, reptile breeders and those working in the industry designing, manufacturing, selling and promoting products for the reptile trade. The writing style in many chapters might be disconcerting for non-academics, with its emphasis on citation of scientific references, but an objective, evidence-based approach is essential for credibility.

There is insufficient space sadly to review each and every chapter, but here are some of the highlights.

## Chapter 10 (Controlled Deprivation and Enrichment, by Robert Mendyk and Lauren Augustine)

I start with this chapter, because it is central to the book in more ways than one. Readers might even benefit from reading this chapter first, as its holistic approach covers many topics which are examined in greater detail elsewhere in the book. In this chapter, the authors explore the concept of “controlled deprivation” first introduced by Gordon M Burghardt, and ways of mitigating this “deprivation” by identifying natural conditions that are critical to each individual species’ welfare, and then providing them within biologically relevant and innately familiar enriched environments. A logical model for reptile enrichment is described. First, basic biological requirements must be met (e.g. needs for adequate space, microclimate, nutrition, social dynamics). Then, existing deprivations, which are to varying degrees inevitable once an animal is in an artificial environment, need to be identified. These may vary between individual animals as well as between species. Changes can then be made, and enrichments added which promote positive welfare states, in particular allowing the reptile greater self-determination and freedom of choice, and thereby reducing one of the major stresses of captivity.

## Chapters 2 and 3 (Physiology and Functional Anatomy, by Harvey B Lilywhite, and Sensory Systems, by Jenna M Crowe-Riddell and Harvey B Lilywhite)

These remain among the best reviews of reptile biology I have ever read, with comprehensive coverage of both structure and function, and with detailed explanations relating these to reptilian physical needs, perception and behaviour. These chapters are also written and illustrated in a very clear style, making the material accessible to those with no formal background in zoology, so could be recommended as essential reading for anyone involved in reptile husbandry.

## Chapter 5 (Normal Behaviour, by James C Gillingham and David L Clark)

Likewise, this is a superb review, in this case of reptilian behaviour across all taxa, well-illustrated with examples from field studies, enabling, in addition, a clear perception of the restrictions on normal behaviour all too often imposed on captive animals by inadequate environments and poor husbandry, which may result in chronic stress.

## Chapters 6 and 7 (Social Behaviour as a Challenge for Welfare, by J Sean Doody and Brains, Behaviour and Cognition: Multiple Misconceptions, by Enrique Font, Gordon M Burghardt, and Manuel Leal)

Over the last 50 years, we have gathered vast amounts of data on sociality in reptiles, which clearly show reptiles as “being capable of sophisticated and complex social interactions.” Doody discusses the difficulties facing reptiles in captivity, ranging from the stresses of isolation for social species to the problems raised by inadequate housing for groups, where territorial behaviour and even inadequate “personal space” may lead to chronic stress or aggressive encounters, even mortality. Chapter 7 offers an unusual but effective approach to appreciation of reptilian intelligence and complex behaviour patterns, by looking at a huge range of studies over the last 20 years or so which sweep away all outdated notions of the reptilian brain being “inferior” in structure and function to that of other vertebrates.

## Chapter 4 (Biology of Stress by Eric J Gangloff and Neil Greenberg)

This offers a detailed account of physiological stress systems, and so would seem an appropriate accompaniment to the abovementioned chapters. However, “stress” is, as the authors point out, “notoriously difficult to define.” A stress response may be a normal and essential part of a healthy life, driving adaptive responses to changing situations; however, if stress is prolonged or intense, it may exceed the animal’s ability to cope. Individuals within a species may also vary widely in their ability to cope, depending on not just the stressor, but their own developmental, ecological, evolutionary, and physiological status.

## Chapter 12 (Ethologically Informed Design and DEEP Ethology in Theory and Practice by Neil Greenberg)

The previous points are discussed further by Neil Greenberg in this chapter, although readers unfamiliar with ethological concepts and vocabulary may find this a bit of a challenge to follow – I certainly did.

## Chapter 8 (Psychological and Behavioural Principles and Problems by Clifford Warwick)

This is largely a review of the consequences of bad husbandry, in particular inadequate housing and unsuitable environmental parameters. Warwick describes two typical behavioural responses: “first, exploratory, search, and escape behaviours; and second, biological shut-down behaviours to withdraw from their surroundings.” However, a table of “behavioural signs of stress or captivity stress” contains numerous behaviours associated with acute defensive and fear responses (e.g. cloacal evacuation, death-feigning, flattened body posture, freezing, hissing) which are more suggestive of an animal’s normal response to a perceived immediate threat (such as an expectation of inhumane handling), rather than the chronic stress of captivity in an unsuitable environment. “Biological shut-down behaviours” characterised by inactivity and disinterest in surroundings (resembling “boredom”) are in my experience much more often indicators of captivity stress in pet reptiles when kept in deficient and inappropriate environments.

## Chapter 13 (Spatial and Thermal Factors, by Phillip C Arena and Clifford Warwick)

This is an especially important chapter owing to the continuing and often hostile debates that persist in many circles, regarding the negative implications of inadequate space and inappropriate furnishing of that space. Appropriate thermal gradients throughout the habitat and the space required to achieve such gradients are not well understood by many reptile keepers and breeders. The latter, in particular, may wish to house large numbers of animals in facilities where space is at a premium. Some have bred many generations of “apparently healthy” reptiles, housed like battery hens, in tiny enclosures such as plastic tubs and snake racks with only the provisions required for basic survival. It is not surprising that people resist change if they believe their husbandry to be adequate; they may perceive demands for improved welfare as a threat to their beliefs as well as their livelihood. Such attitudes towards reptile-keeping resemble those that were shown by many towards intensive poultry farming – but these attitudes are being changed, mainly by evidence of the positive effects of improved welfare. Education is vital as to the welfare benefits of offering enough space for self-determination and expression of natural behaviours, as well as the health benefits of an adequate range of thermal zones for thermoregulatory choices. The authors present a powerful case. It is disappointing, however, that the importance of species-appropriate full spectrum lighting in the creation of these thermal zones is not mentioned. Research is increasingly revealing the benefits of all wavelengths found in natural sunlight. Irradiance levels can have profound effects on behaviour and circadian rhythms. Moreover, short-wavelength infrared is biologically active, not just a heat source.

## Chapter 14 (Nutritional Considerations, by Michael Thomas Maslanka, Fredric L Frye, Barbara Ann Henry, and Lauren Augustine)

Gratifyingly this chapter which follows on does discuss the importance of UVB provision as well as topics such as a review of the entire process of food selection by the reptile, its passage through the digestive system and the excretion of waste products. Target nutrient values for various diets are discussed, as are requirements for water, and the effects of stress, improper diets, obesity and starvation.

## Chapter 16 (Evidential Thresholds for Species Suitability in Captivity, by Mike Jessop, Anthony Pilny, Clifford Warwick and Martin Whitehead)

The theme running throughout the book, as one might expect from the title, is that when reptiles are kept in captivity, this confinement in an artificial environment is inevitably a deprivation. However, it is a “controlled deprivation” – the keeper is in control, and bears the responsibility of identifying problems and tackling issues with the best welfare outcome for the animal always in mind. This positive approach is extremely encouraging – that by understanding the reptile, its needs, its strengths and weaknesses, and its world view, we can improve husbandry and work towards giving these amazing animals “a life worth living”. I approached this review with a degree of apprehension, knowing the connection of some authors with the Animal Rights movement, who openly admit to an agenda of eliminating all reptile-keeping. Such negative views are not helpful in encouraging improvements in animal welfare and their absence from almost all chapters is a relief. However, I do have reservations about some of the material in chapter 16. It cannot be denied that many pet reptiles are kept in very unsuitable conditions, largely owing to owners’ ignorance of their needs or – sadly – due to misinformation gathered with the best of intentions from widely available published resources, social media, so-called “experts” and “influencers” and even pet product manufacturers. It is also obvious to those of us involved in reptile rehabilitation, rescue and veterinary care that no reptiles are easy to care for, since even their ectothermic nature requires environmental control for their entire lives, and many have far more specialised requirements and can live much longer than many owners expect. The authors usefully review the current models for assessing welfare: the “Five Freedoms”, the “Five Domains” (Mellor 2016, 2017) and the RSPCA’s “Five Welfare Needs.” However, instead of simply stating the obvious, that all reptiles could be considered difficult to keep, with some species extremely difficult to keep, the authors have devised an overly complicated “EMODE system” which allocates points to rate the suitability of a particular reptile for captive care. All species of reptiles are pre-determined to be “moderately” difficult. This seems disingenuous since each species is then scored for features thought to make them even less suitable to keep, including such basic things as small size, large size, lifespan over 10 years and nocturnality. Possessing just one of these features puts the animal straight into the “difficult to keep” category. It is obvious that the EMODE system has been designed to categorise *all* reptiles as either difficult or extremely difficult, as all but one of the worked examples in the appendix demonstrate. That one exception, however, has been scored incorrectly. Even less edifying is the second part of the EMODE system which seeks to score humans as regards their suitability to keep a reptile. Unless you have professional training or detailed husbandry experience, or can “identify at least 40 welfare-related signs” you are likely to have “Low” suitability to keep a reptile; and if there are any elderly persons or children under five years in your “extended circle” you are cautioned not to keep reptiles at all. What hope for our future herpetologists, zoologists and animal welfare experts? Most of us developed our love of animals and dedication to our profession through keeping pets through our formative years.

That said, the authors’ concern over large numbers of reptiles kept in unsuitable conditions is warranted. Therefore, a degree of restriction on ownership without evidence of a suitable level of understanding of the animal’s needs, and of the owner’s ability to fulfil those needs, would appear long overdue. Banning ownership is not the answer and would have disastrous effects on welfare as reptile keeping would assuredly go underground. Some form of licensing following completion of a recognised training program, for example, might be one positive way forward. Admittedly there are no easy answers.

Education geared towards a reminder that no reptiles are easy to keep, and provision of high quality advice and assistance for those who do undertake ownership seriously, would go a long way towards the goal of providing reptiles with “a life worth living.” I hope this book enables readers to take a step in this direction.
